# The Herbal Blend of *Sphaeranthus indicus* and *Garcinia mangostana* Reduces Adiposity in High-Fat Diet Obese Mice

**DOI:** 10.3390/foods13183013

**Published:** 2024-09-23

**Authors:** Sumin Kang, Hayoon Kim, Chaeyoung Bang, Jung Hyeon Park, Gwang-woong Go

**Affiliations:** 1 Department of Food and Nutrition, Hanyang University, Seoul 04763, Republic of Korea; sumin.kang708@gmail.com (S.K.); kimhayoon0408@gmail.com (H.K.); 2 Green Store Inc., R&D Center, Seoul 08501, Republic of Korea; joybang@green-store.co.kr (C.B.); jhpark04@green-store.co.kr (J.H.P.)

**Keywords:** Meratrim, *Sphaeranthus indicus*, *Garcinia mangostana*, obesity, de novo lipogenesis, non-shivering thermogenesis

## Abstract

Obesity is swiftly becoming a global epidemic, leading to numerous metabolic disorders and substantial socio-economic burdens. Investigating natural bioactive compounds is crucial to support the use of traditional anti-obesity medications while mitigating the adverse effects. This study posited that a combination of *Sphaeranthus indicus* and *Garcinia mangostana* (Meratrim) could prevent fat accumulation in obese mice. We used 4-week-old C57BL/6NTac mice, dividing them into six groups: (1) normal diet (ND); (2) high-fat diet (HFD, 45% kcal from fat); (3–5) Meratrim150, Meratrim300, and Meratrim450 (HFD with 150, 300, and 450 mg/kg bw of Meratrim); and (6) Metformin (HFD with 150 mg/kg bw of metformin). Meratrim was administered orally each day for 20 weeks. The group receiving 450 mg/kg of Meratrim showed a significant reduction in body weight and fat mass without changes in food consumption. The Meratrim450 group had markedly lower triglyceride levels in both serum and liver. Importantly, Meratrim-supplemented mice improved lipid homeostasis by inhibiting hepatic de novo lipogenesis and activating energy catabolic pathways such as non-shivering thermogenesis in brown adipose tissue. Our results suggest that the herbal mixture of *Sphaeranthus indicus* and *Garcinia mangostana* (Meratrim) is a promising natural anti-obesity agent, owing to its efficacy in reducing body fat and enhancing lipid homeostasis.

## 1. Introduction

Excess fat accumulation, globally, regionally, or in organs, as ectopic lipids leads to obesity [1]. Obesity is escalating globally to epidemic levels; approximately 16% of adults aged 18 and older worldwide were classified as obese in 2022, indicating a more than two-fold rise in obesity prevalence from 1990 to 2022 [2]. Obesity, particularly the excessive accumulation of ectopic fat, accelerates the progression of diverse metabolic diseases such as type 2 diabetes mellitus, cardiovascular disease, non-alcoholic fatty liver disease, and dyslipidemia [3,4]. Alongside these metabolic disorders, obesity is regarded as a health concern that not only shortens individuals’ life spans but results in substantial economic losses on a societal level [5,6]. Several medications, such as orlistat and phentermine, are widely used in the treatment of obesity. Recently, incretin-based drugs like semaglutide and tirzepatide have garnered attention as potential long-term pharmacotherapy options for obesity. However, these anti-obesity drugs have yet to address persistent side effects, including drug abuse and dependence, headaches, elevated blood pressure, and depression [7]. Natural bioactive compounds are considered promising solutions for minimizing the adverse effects of anti-obesity medications and improve the sustainability of obesity treatment [8]. Herbal medicine, deeply rooted in traditional healing systems across cultures worldwide, is currently experiencing renewed interest in exploring the therapeutic potential of herbal extracts for combating obesity [9]. Moreover, herbal medicines are typically formulated as blends of multiple herbs rather than prescribing single herbs to patients. The efficacy of mixed herbal formulations depends on the interactions among the various herbs [10]. Specifically, the constituent herbs synergistically work together within a mixed herbal formulation to produce beneficial effects. In contrast, any potential adverse side effects of individual components are mitigated by the presence of others [11].

*Sphaeranthus indicus* is a traditional medicinal herb found in southern India. It contains bioactive components, including 7-hydroxyfrulloanolide from the flower, which are known for their antioxidant, anti-inflammatory, and anti-hyperglycemic effects [12,13,14]. *Garcinia mangostana* is a fruit indigenous to Asia with several biological polyphenolic compounds, including α-mangostin, which have demonstrated beneficial effects such as anti-obesity, anti-diabetic, and anti-inflammatory properties [15,16,17]. The combined herbal blend of *Sphaeranthus indicus* and *Garcinia mangostana* has been clinically validated to reduce body weight [18,19]. However, the specific molecular mechanism behind its weight loss efficacy remains unclear.

Therefore, this study aims to investigate the anti-obesity effect of the herbal blend of *Sphaeranthus indicus* and *Garcinia mangostana* (Meratrim) in high-fat-diet-induced obese C57BL/6NTac mice.

## 2. Materials and Methods

### 2.1. Herbal Blend Preparation

The dried flower heads of *Sphaeranthus indicus* were pulverized into a coarse powder and subjected to extraction with 90–95% ethanol under ambient conditions. The resulting extract was filtered, concentrated under a vacuum at a temperature not exceeding 60 °C, and then subjected to liquid–liquid extraction using ethyl acetate. The ethyl acetate phase was concentrated to obtain a thick paste. Similarly, the dried fruit rinds of *Garcinia mangostana* were pulverized into a coarse powder and extracted with 80–90% ethanol at 70–80 °C. The extract was filtered, concentrated under vacuum at a temperature not exceeding 70 °C, and then dried under a vacuum at 65–75 °C to obtain a dry powder. The extracts of *Sphaeranthus indicus* and *Garcinia mangostana* were then blended in a 3:1 ratio, along with excipients, and the mixture was dried and powdered to formulate the final product, Meratrim. The Drug Extract Ratio (DER) for *Sphaeranthus indicus* was determined to be 20–21:1, indicating that 20 to 21 parts of the raw material were used to produce 1 part of the final extract. Similarly, the DER for *Garcinia mangostana* was found to be 9–12:1, demonstrating that 9 to 12 parts of the fruit rind were required to yield 1 part of the final extract. The final product, Meratrim, contained 37.5% active substances from *Sphaeranthus indicus* and 12.5% from *Garcinia mangostana*.

### 2.2. Animal Experiment

The Institutional Animal Care and Use Committee (IACUC) of Hanyang University approved all animal experiment procedures (HY-IACUC-2022-0127). Four-week-old male C57BL/6NTac mice were purchased from Daehan BioLink (Seoul, Republic of Korea). The animal facility was maintained at 21 ± 1 °C and 50 ± 10% relative humidity, with a 12:12 h light–dark cycle. Five mice per cage, housed in a ventilated cage, were acclimatized for 1 week before experimentation. A total of 60 mice were randomly assigned into six groups (*n* = 10): (1) normal diet (ND, 13% kcal from fat), (2) high-fat diet (HFD, 45% kcal from fat, negative control), (3) Meratrim150 (HFD with 150 mg/kg bw Meratrim), (4) Meratrim300 (HFD with 300 mg/kg bw Meratrim), (5) Meratrim450 (HFD with 450 mg/kg bw Meratrim), and (6) Metformin (HFD with 150 mg/kg bw metformin, positive control). The combined extract of *Sphaeranthus indicus* and *Garcinia mangostana* (Meratrim) was dissolved in 10% Kolliphor^®^ EL (BASF, Ludwigshafen, Germany), and 0.9% saline solution was orally gavaged daily for 20 weeks.

### 2.3. Growth Performance and Body Composition

Body weight and feed intake were monitored weekly for 20 weeks. The feed efficiency ratio (FER) was calculated as the weight gain (g/day)/feed intake (g/day) × 100. At the end of the intervention, mice were anesthetized by intraperitoneal injection using a mixture of ketamine (100 mg/kg bw) and xylazine (10 mg/kg bw). Body composition, including fat mass, lean mass, fat in tissue, and bone mineral density (BMD), was measured using dual-energy X-ray absorptiometry (DEXA, Medikors, Sungnam, Republic of Korea).

### 2.4. Blood Biochemical Analysis

At the end of the intervention, the blood was collected through the retro-orbital cavity of mice after 6 h of fasting. The serum was separated using a centrifuge (2000× *g*, 15 min, 4 °C) and stored at −80 °C until further analysis. Aspartate aminotransferase (AST) and alanine aminotransferase (ALT) levels were examined using commercial kits (Asan Pharmaceutical, Seoul, Republic of Korea) to assess liver toxicity. To evaluate serum lipid profiles, serum triglyceride and total cholesterol levels were measured using commercial kits (Wako, Osaka, Japan). All commercial assay kits were performed according to the manufacturer’s instructions.

### 2.5. Hepatic Triglycerides

The liver tissue was homogenized with an NP40 lysis buffer (Thermo Fisher Scientific, Waltham, MA, USA) and then exposed to 95 °C to extract lipids. The supernatant was separated using a centrifuge (14,000× *g*, 2 min, 4 °C). According to the manufacturer’s instructions, the hepatic triglyceride extracted from the supernatant was measured using a commercial kit (Wako).

### 2.6. Immunoblotting

The protein of the liver and brown adipose tissue was extracted using RIPA lysis buffer (BIOMAX, Gyeonggi-do, Republic of Korea) complemented with protease and phosphatase inhibitors (Cell Signaling Technology, Dancers, MA, USA). The total protein content was determined using a BCA protein assay kit (Thermo Fisher Scientific, Saint Louis, MO, USA) with bovine serum albumin (BSA) as a standard. Subsequently, the protein lysate samples were mixed with Laemmli sample buffer (Bio-Rad, Hercules, CA, USA) and loaded onto 20% sodium dodecyl sulfate–polyacrylamide gel electrophoresis (SDS-PAGE) gels, followed by transfer to polyvinylidene fluoride (PVDF) membranes. The membranes were blocked with 5% BSA for 50 min at room temperature and then incubated with primary antibodies at 4 °C overnight. The following primary antibodies were used for immunoblotting: acetyl-CoA carboxylase 1 (ACC1, #4190), phosphorylated ACC at serine 79 (ACC1-pS79, #3661), fatty acid synthase (FAS, #3189), stearoyl CoA desaturase 1 (SCD1, #2794), uncoupling protein 1 (UCP1, #14670), peroxisome proliferator-activated receptor-γ coactivator-1α (PGC-1α, #PA5-72948), and glyceraldehyde-3-phosphate dehydrogenase (GAPDH, #2118). GAPDH was used to control its loading. Afterward, the membrane was incubated with the HRP-conjugated secondary antibody (Bio-Rad) for 1 h at room temperature. Proteins were quantified using Image Lab 6.1 software (Bio-Rad).

### 2.7. Statistical Analysis

All data were presented as the mean ± standard error of the mean (SEM). The results were analyzed using one-way ANOVA followed by Tukey’s multiple comparison test (Prism 6, GraphPad, San Diego, CA, USA). A *p* < 0.05 was considered statistically significant.

## 3. Results

### 3.1. Meratrim Administration Reduced Body Weight without Altering Feed Intake

**Figure 1:**
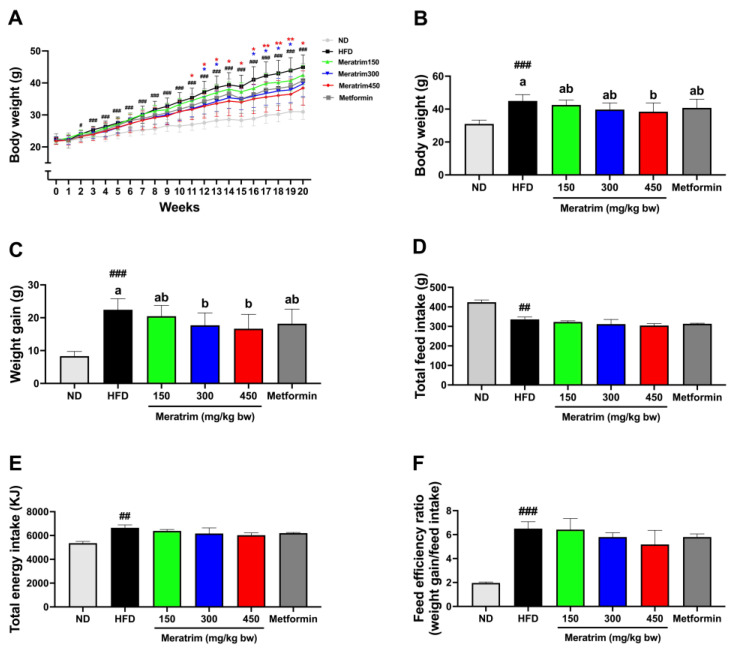
Meratrim administration reduced body weight and weight gain without altering feed intake. (**A**) Body weight changes over 20 weeks, (**B**) body weight at 20th week, (**C**) weight gain, (**D**) total feed intake, (**E**) total energy intake, and (**F**) feed efficiency ratio of ND, HFD, metformin-treated, and Meratrim-treated mice. Values are expressed as mean ± SEM. ^#^ Significant differences were analyzed using an unpaired *t*-test between ND and HFD (^##^ *p* < 0.01 and ^###^ *p* < 0.001). * Significant differences were analyzed using one-way ANOVA with Tukey’s multiple comparisons between groups (* *p* < 0.05 and ** *p* < 0.01) ^a–b^ Significant differences were analyzed using one-way ANOVA with Tukey’s multiple comparisons between groups (*p* < 0.05). ND, normal diet; HFD, high-fat diet; Meratrim, combined extract of Sphaeranthus indicus and Garcinia mangostana.

We generated obese mice using a 45% high-fat diet for 20 weeks, considering that the typical American or European diet comprises approximately 36~40% fat by energy [20]. After 2 weeks of a 45% high-fat diet supplement, the high-fat diet group showed elevated body weight compared to the normal diet group, confirming the successful establishment of diet-induced obese mice. We measured the body weight, weight gain, feed intake, and feed efficiency ratio weekly to assess the anti-obesity effect of Meratrim (Figure 1). The initial body weights of all groups were assigned without significant differences (22.25 ± 0.4 g). From 11 weeks of intervention onwards, the Meratrim450 group consistently recorded lower body weight compared to the control group, culminating in a 14.6% decrease in body weight by the end of the intervention (*p* < 0.05) (Figure 1A,B). Although not statistically significant, the Meratrim150 and Meratrim300 groups also recorded weight reductions of 5.49% (*p* = 0.74) and 11.6% (*p* = 0.06), respectively. (Figure 1B). Similarly, the administration of 300 and 450 mg/kg bw Meratrim resulted in 21.0% and 25.8% lower weight gain, respectively, compared to the control group (*p* < 0.05) (Figure 1C). We found there was no significant difference in total feed intake and total energy intake among the high-fat diet groups (Figure 1D,E), verifying the weight loss effect of Meratrim without altering appetite. As shown in Figure 1F, all high-fat diet groups exhibited a higher feed efficiency ratio (FER) than the normal diet group. While the Meratrim450 group did not show a significant reduction, it did record a 20.4% lower FER, indicating the Meratrim450 group exhibited an effect in preventing weight gain induced by a high-fat diet. Our findings suggested Meratrim has weight reduction potential. Specifically, the administration of 450 mg/kg bw Meratrim effectively improved weight loss phenotypes in high-fat-diet-induced obese mice.

### 3.2. Meratrim Administration Mitigates Body Fat Accumulation

**Figure 2:**
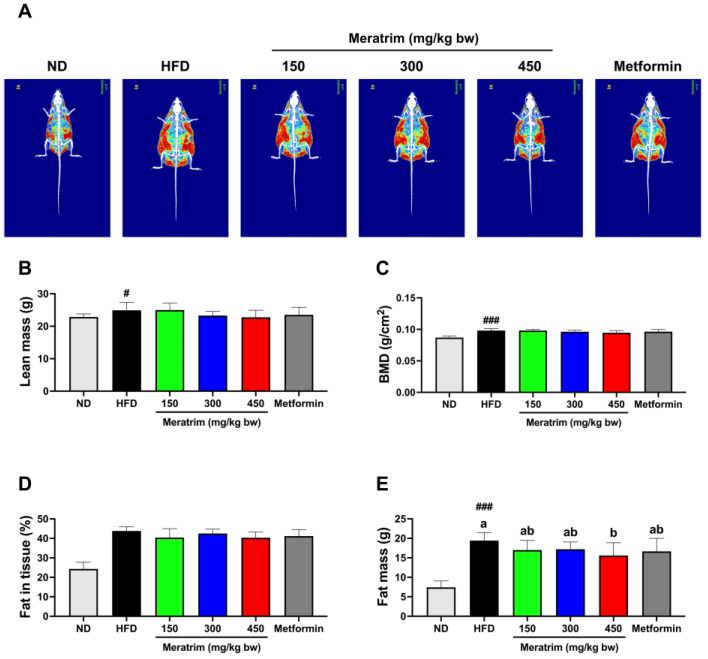
Meratrim administration reduced body fat mass in high-fat-diet-induced obese mice. (**A**) Image of DEXA, (**B**) lean mass, (**C**) BMD, (**D**) fat in tissue, and (**E**) fat mass. Values are expressed as mean ± SEM. ^#^ Significant differences were analyzed using an unpaired *t*-test between ND and HFD (^#^ *p* < 0.05 and ^###^ *p* < 0.001). ^a–b^ Significant differences were analyzed using one-way ANOVA with Tukey’s multiple comparisons between groups (*p* < 0.05). ND, normal diet; HFD, high-fat diet; Meratrim, combined extract of Sphaeranthus indicus and Garcinia mangostana.

DEXA, considered a highly reliable method to measure fat content in in vivo models, allows for the non-invasive and precise analysis of body composition, including body fat, lean mass, and BMD [21]. We conducted DEXA at the end of the intervention to assess the body composition changes by Meratrim supplementation. The red and green portions of the image represent fat and lean tissue, respectively (Figure 2A). There was no significant difference in lean mass and BMD among high-fat diet groups (Figure 2B,C). Although we did not observe a significant change in fat in tissue, the Meratrim450 group showed a 8.88% reduction compared to the control group (*p* = 0.10) (Figure 2D). Meanwhile, the fat mass of the Meratrim450 group significantly decreased by 19.8% (*p* < 0.05) (Figure 2E). These results indicated the body weight loss observed with the administration of 450 mg/kg bw Meratrim was primarily due to a reduction in fat mass.

### 3.3. Meratrim Administration Improved Lipid Profiles without Hepatotoxicity

**Figure 3:**
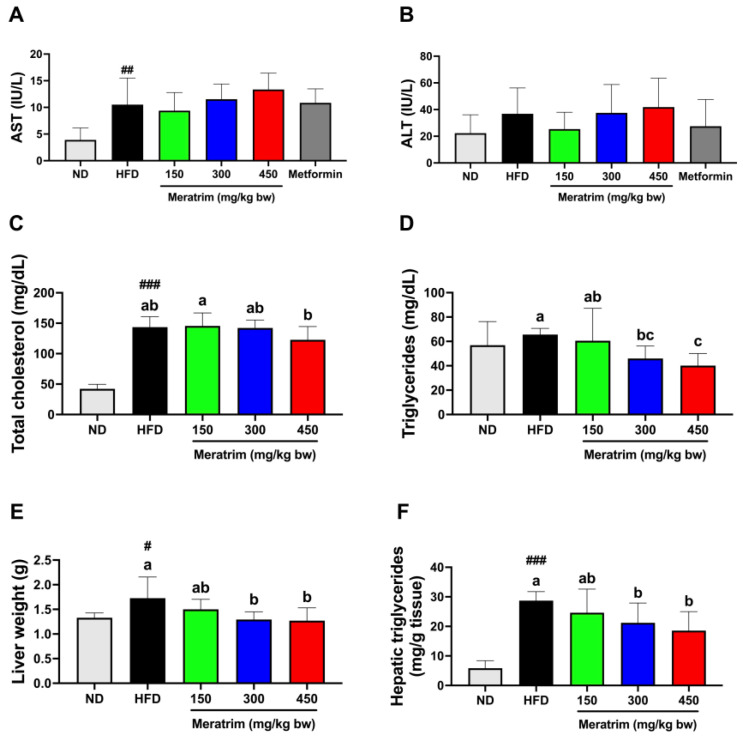
Meratrim administration alleviates lipid profiles in high-fat-diet-induced obese mice. Analysis of hepatotoxicity indicators including (**A**) AST and (**B**) ALT in serum of ND, HFD, metformin-treated, and Meratrim-treated mice. (**C**) Serum total cholesterol and (**D**) triglycerides were measured in the serum of each group. (**E**) Liver weight and (**F**) hepatic triglycerides were measured in the liver tissue of each group. Values are expressed as mean ± SEM. ^#^ Significant differences were analyzed using an unpaired *t*-test between ND and HFD (^#^ *p* < 0.05, ^##^ *p* < 0.01, and ^###^ *p* < 0.001). ^a–c^ Significant differences were analyzed using one-way ANOVA with Tukey’s multiple comparisons between groups (*p* < 0.05). ND, normal diet; HFD, high-fat diet; Meratrim, combined extract of Sphaeranthus indicus and Garcinia mangostana.

We measured serum levels of AST and ALT to assess whether Meratrim induces hepatotoxicity. Elevated AST and ALT levels are not only utilized as indicators of hepatotoxicity but also serve as diagnostic markers for non-alcoholic fatty liver disease (NAFLD) [22,23]. It is known that a high-fat diet increases AST and ALT levels [24]. In C57BL/6NTac mice aged 90 to 135 days, the median levels of AST and ALT are reported to be 52 and 27 (IU/L), respectively [25]. There were no significant elevations in AST and ALT levels among high-fat diet groups (Figure 3A,B). Moreover, the AST:ALT ratio, used as a marker for various liver diseases, was recorded as less than one in all groups. This further supports the evidence that significant hepatotoxicity due to Meratrim administration was not observed.

Obesity resulting from a high-fat diet often leads to high levels of triglycerides and LDL cholesterol in the blood, increasing the risk of dyslipidemia and cardiovascular risk factors [26,27]. Meratrim-supplemented mice did not show any changes in total cholesterol levels compared to the control group (Figure 3C). However, the serum triglycerides of Meratrim300 and Meratrim450 groups were significantly decreased, by 31.3% and 40.0%, respectively (*p* < 0.01 and *p* < 0.001, respectively) (Figure 3D).

Accordingly, we observed a reduction in liver weight of 300 and 450 mg/kg bw Meratrim-supplemented mice (*p* < 0.05) (Figure 3E). An increase in liver weight can be attributed to various factors such as the accumulation of lipids, glycogen, or other substances within tissue sections [28]. We measured the hepatic triglyceride content to substantiate that the decrease in liver weight results from reduced hepatic lipid accumulation. Consistent with the reduction in liver weight, the hepatic triglyceride levels of the Meratrim300 and Meratrim450 groups declined compared to the control group (*p* < 0.05 and *p* < 0.01, respectively) (Figure 3F). Taken together, the administration of Meratrim showed a significant reduction in serum triglycerides, liver weight, and hepatic triglycerides levels in obese mice. These findings suggest that Meratrim could be an effective intervention for mitigating the metabolic disturbances, including dyslipidemia and NAFLD, associated with high-fat-diet-induced obesity.

### 3.4. Hepatic De Novo Lipogenesis Was Inhibited by Meratrim Administration

Hepatic de novo lipogenesis (DNL), activated by high-fat and high-carbohydrate diets, synthesizes fatty acids from excess carbohydrates [29]. Excessive hepatic lipogenesis induced by obesity leads to the development of NAFLD and, subsequently, to metabolic diseases such as cardiovascular diseases, dyslipidemia, and type 2 diabetes [30]. Accordingly, the inhibition of hepatic DNL could serve as an effective therapeutic target for obesity and metabolic disorders [31]. ACC1 catalyzes the rate-limiting step of DNL by converting acetyl-CoA into malonyl-CoA, which serves as both a substrate for lipogenesis and as an inhibitor of fatty acid oxidation [32,33]. Indeed, the phosphorylation of serine 79 on ACC1, mediated by AMP-activated protein kinase (AMPK), inhibits malonyl-CoA production [34]. FAS and SCD1 also play vital roles as enzymes in hepatic DNL [35]. Fatty acids, including palmitate, are synthesized from malonyl-CoA by FAS. Subsequently, SCD1 catalyzes the production of monounsaturated fatty acids [36,37]. 

**Figure 4:**
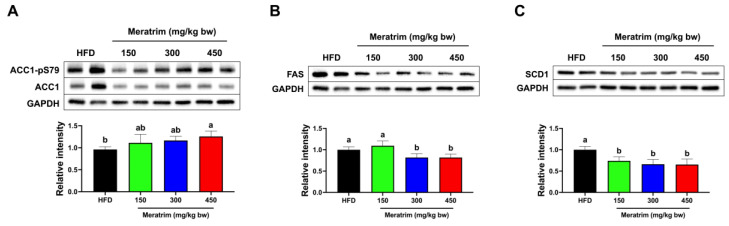
Meratrim decreased hepatic de novo lipogenesis-related enzymes in the liver of high-fat-diet-induced obese mice. Protein expression of (**A**) ACC1-pS79 and ACC1, (**B**) FAS, and (**C**) SCD1 in the liver of HFD and Meratrim-treated mice. Values are expressed as mean ± SEM. ^a–b^ Significant differences were analyzed using one-way ANOVA with Tukey’s multiple comparisons between groups (*p* < 0.05). HFD, high-fat diet; Meratrim, combined extract of Sphaeranthus indicus and Garcinia mangostana.

Therefore, based on the decline in hepatic triglyceride levels following Meratrim administration, we monitored the changes in the expression of lipogenesis-related enzymes within the liver tissue (Figure 4). The administration of Meratrim diminished ACC1 activation by increasing phosphorylated ACC1-pS79, especially with the Meratrim450 group showing a significant increase of 25% (*p* < 0.05) (Figure 4A). In addition, there was an approximately 30% decrease in the expression of FAS observed in both the Meratrim300 and Meratrim450 groups (Figure 4B) (*p* < 0.05). The hepatic expression of SCD1 was significantly lower in the Meratrim-treated groups compared to the control group. Specifically, the administration of 450 mg/kg bw of Meratrim induced a reduction of 35% (*p* < 0.01) (Figure 4C). Accordingly, these findings indicate that Meratrim supplement can contribute to improving hepatic steatosis and obesity-related metabolic disorders by inhibiting the hepatic DNL pathway.

### 3.5. Non-Shivering Thermogenesis in Brown Adipose Tissue Was Enhanced by Meratrim Administration

**Figure 5:**
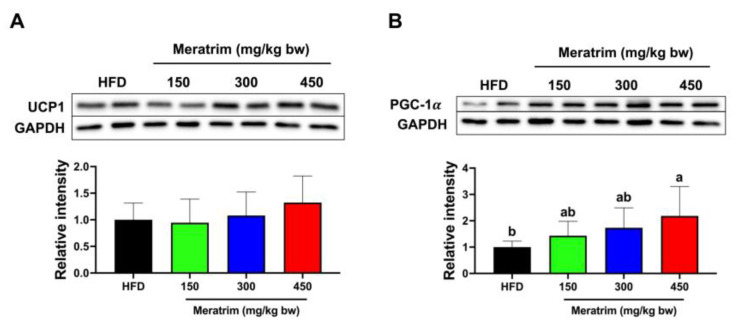
Meratrim increased thermogenic-related enzymes in the brown adipose tissue of high-fat-diet-induced obese mice. Protein expression of (**A**) UCP1 and (**B**) PGC-1α in the brown adipose tissue of HFD and Meratrim-treated mice. Values are expressed as mean ± SEM. ^a–b^ Significant differences were analyzed using one-way ANOVA with Tukey’s multiple comparisons between groups (*p* < 0.05). HFD, high-fat diet; Meratrim, combined extract of Sphaeranthus indicus and Garcinia mangostana.

Non-shivering thermogenesis is a metabolic process that generates heat in response to cold in the brown adipose tissue. Energy expenditure and heat production through non-shivering thermogenesis are potential strategies for combating obesity and obesity-related diseases [38,39]. The primary mechanism of non-shivering thermogenesis involves mitochondrial uncoupling mediated by UCP1, a specific protein found in the inner membrane of brown adipose tissue mitochondria [40]. PGC-1α is another critical regulator of thermogenesis by upregulating mitochondrial biogenesis in brown adipose tissue [41]. Thus, we evaluated the expression of the thermogenic-related proteins, UCP1 and PGC-1α, in brown adipose tissue (Figure 5). Although there was no significant change, mice supplemented with 450 mg/kg bw of Meratrim showed a 32.4% increase in UCP1 expression (Figure 5A). Importantly, PGC-1α, the master regulator of mitochondrial biogenesis, was enhanced in the Meratrim-treated groups (Figure 5B). Furthermore, the Meratrim450 group particularly improved the expression of PGC-1α by 118% (*p* < 0.01). Hence, these results suggest that Meratrim administration ameliorates obesity-related metabolic disorders by enhancing energy expenditure via thermogenesis in brown adipose tissue.

## 4. Discussion

Obesity, characterized by excessive adiposity, is rapidly becoming a global epidemic, contributing to various metabolic disorders and significant societal economic loss [2,42]. Due to the limitations of conventional anti-obesity drugs, such as their low accessibility and negative side effects, the need for the development of natural bioactive agents to manage obesity is being emphasized [43]. In particular, combining multiple herbal products could synergistically enhance their anti-obesity impact on numerous targets [44]. Building on this concept, several clinical studies have previously examined the efficacy of a herbal formulation composed of *Sphaeranthus indicus* and *Garcinia mangostana* for weight management [18,19,45]. However, the molecular basis of its anti-obesity effects has only been described in cellular in vitro models. Therefore, we assessed the ability of a herbal blend of *Sphaeranthus indicus* and *Garcinia mangostana* to improve obesity in high-fat-diet-induced obese mice.

The results of this study demonstrated that the administration of the combined extract of *Sphaeranthus indicus* and *Garcinia mangostana* (Meratrim) effectively reduced adiposity and improved lipid homeostasis. We observed that the administration of 450 mg/kg bw Meratrim resulted in a notable body weight reduction, with the Meratrim450-treated group showing an approximately 13.3% lower body weight compared to the control group at the end of the intervention. According to prior studies, *Garcinia mangostana* showed a weight loss effect in obese rat models. The supplementation of a high fat diet with 5% *Garcinia mangostana* rind effectively reduced body weight, feed efficiency, and fat mass [46]. In addition, the oral administration of *Garcinia mangostana* flesh at doses of 200, 400, and 600 mg/kg bw dramatically decreased body weight and weight gain [47]. Consistent with our findings, the weight reduction efficacy was exhibited in obese human subjects with a body mass index (BMI) between 30 and 40 kg/m^2^ who received 400 mg of the herbal blend of *Sphaeranthus indicus* and *Garcinia mangostana* twice daily [18,45]. Furthermore, a significant reduction in body weight, by supplementing with this herbal blend, was demonstrated in healthy, overweight human subjects with a mean BMI of 28.3 kg/m^2^ [19]. Although these previous clinical trials did not provide body composition data using DEXA, they showed a significant reduction in waist size, hip size, and waist-to-hip ratio (WHR), which can be used to measure visceral fat [48]. In sum, our study verified that the obvious weight loss following Meratrim administration was due to decreased adiposity, as measured by DEXA.

In addition, we examined the impact of the herbal formulation of Meratrim on serum and hepatic triglycerides, along with liver function markers. The obese and overweight human subjects receiving 400 mg of *Sphaeranthus indicus* and *Garcinia mangostana* twice daily presented a significant decrease in serum triglycerides, LDL cholesterol, and total cholesterol levels without impairment of liver function [18,19]. Indeed, *Sphaeranthus indicus* and *Garcinia mangostana* have each been reported to have activities that improve dyslipidemia and alleviate liver damage. The 400 and 800 mg/kg of methanolic extracts of *Sphaeranthus indicus* whole plants are shown to suppress serum triglyceride levels in dexamethasone-induced insulin-resistant mice [49]. Similarly, treatment with 250 or 500 mg/kg of alcoholic extract of *Sphaeranthus indicus* diminished serum triglyceride and total cholesterol and restored HDL cholesterol in streptozotocin–nicotinamide diabetic rats [50]. Likewise, obese mice fed with 50 mg/kg bw α-mangostin have been reported to have reduced serum lipid profiles, including cholesterol and triglycerides [51]. Furthermore, *Garcinia mangostana* pericarp extract (40, 200, and 1000 mg/kg/day) significantly improved dextran sulfate sodium (DSS)-induced hepatic injury [52], and α-mangostin also decreased serum AST and ALT levels in acetaminophen-induced hepatotoxicity in mice [53]. Based on these previous findings, our study demonstrated that mice treated with 300 and 450 mg/kg bw of Meratrim had significantly reduced serum and hepatic triglyceride levels. Moreover, there was no significant change in serum AST and ALT levels, indicating that its activity in improving lipid profiles is not accompanied by hepatotoxicity. 

In accordance with the improvement in the obese phenotype and triglyceride profiles, we further observed significant changes in the liver tissue’s key lipogenic pathway enzymes. An increase in DNL enzymes plays a pivotal role in expanding fat mass, suggesting that the inhibition of lipogenic enzymes could mitigate the progression of obesity [29]. Our research proved the administration of 450 mg/kg bw Meratrim suppresses the activity of ACC1 by phosphorylating serine 79. Subsequently, Meratrim treatment also inhibited the expression of FAS and SCD1. Actually, it has previously been reported that *Garcinia mangostana* and its bioactive compounds suppress hepatic steatosis through Sirtuin1 (Sirt1)-AMPK in high-fat-diet-induced obese mice [51,54]. Further fatty acid synthesis genes such as SREBP1c, lipoprotein lipase (LPL), and SCD1 were also reduced by α-mangostin in obese mice [55]. Similar to our research, treatment with 10 µg/mL of a herbal blend of *Sphaeranthus indicus* and *Garcinia mangostana* inhibited FAS in 3T3-L1 adipocytes and increased the phosphorylation of ACC 1 at serine 79 in HepG2 hepatocytes [19]. In another study, 5, 10, and 15 µg/mL of this blend showed a dose-dependent downregulation of adipogenic enzymes, including peroxisome proliferator-activated receptor gamma (PPARγ), adipose differentiation-related protein (ADRP), and a cluster of differentiation 36 (CD36) in 3T3-L1 adipocytes [18]. Collectively, these findings support the idea that the adiposity-reducing property of Meratrim is attributed to it inhibiting the DNL pathway in the liver tissue.

Interestingly, we confirmed the additional impact of Meratrim on brown adipose tissue. Activating non-shivering thermogenesis is regarded as an effective therapeutic strategy for combating obesity. In this study, we investigated whether Meratrim might enhance the expression of UCP1, a specific uncoupling protein found in brown adipose tissue. Although the increments observed following Meratrim treatment are not statistically significant, the trends suggest that Meratrim may help activate energy expenditure by upregulating UCP1. Notably, our result demonstrated that Meratrim particularly augments PGC-1α, indicating that Meratrim activates mitochondrial biogenesis, further influencing energy expenditure. Our observations suggest that Meratrim can also mitigate obesity by inhibiting the DNL pathway and improving non-shivering thermogenesis.

## 5. Conclusions

Our study demonstrated that the combined extract of *Sphaeranthus indicus* and *Garcinia mangostana* (Meratrim) effectively reduced body weight and improved metabolic health in high-fat-diet-induced obese mice. The administration of 450 mg/kg bw Meratrim significantly lowered body weight and fat mass without altering feed intake, indicating its potential as an anti-obesity agent. Furthermore, Meratrim improved lipid profiles, reducing serum and hepatic triglyceride levels without causing hepatotoxicity. These beneficial effects were attributed to hepatic de novo lipogenesis inhibition, as evidenced by the downregulation of key lipogenic enzymes, ACC1, FAS, and SCD1. Additionally, Meratrim enhanced non-shivering thermogenesis in brown adipose tissue by upregulating PGC-1α, suggesting increased energy expenditure. Our study stands out from previous research that has primarily focused on in vitro cellular models. This article reveals the dual mechanism of action by which the herbal blend of *Sphaeranthus indicus* and *Garcinia mangostana* reduces adiposity in vivo: through the inhibition of hepatic de novo lipogenesis and the enhancement of non-shivering thermogenesis in brown adipose tissue. Overall, these findings indicate that Meratrim holds promise for developing natural therapeutic agents to combat obesity and its associated metabolic disorders.

## Data Availability

The original contributions presented in the study are included in the article, further inquiries can be directed to the corresponding author.
